# Universal properties of metal-supported oxide films from linear scaling relationships: elucidation of mechanistic origins of strong metal–support interactions†

**DOI:** 10.1039/d2sc06656d

**Published:** 2023-02-04

**Authors:** Kaustubh J. Sawant, Zhenhua Zeng, Jeffrey P. Greeley

**Affiliations:** a Charles D. Davidson School of Chemical Engineering, Purdue University 480 Stadium Mall Drive West Lafayette IN 47907 USA jgreeley@purdue.edu

## Abstract

The properties of ultrathin (1–2 monolayer) (hydroxy)oxide films on transition metal substrates have been extensively studied as models of the celebrated Strong Metal–Support Interaction (SMSI) and related phenomena. However, results from these analyses have been largely system specific, and limited insights into the general principles that govern film/substrate interactions exist. Here, using Density Functional Theory (DFT) calculations, we analyze the stability of ZnO_*x*_H_*y*_ films on transition metal surfaces and show that the formation energies of these films are related to the binding energies of isolated Zn and O atoms *via* linear scaling relationships (SRs). Such relationships have previously been identified for adsorbates on metal surfaces and have been rationalized in terms of bond order conservation (BOC) principles. However, for thin (hydroxy)oxide films, SRs are not governed by standard BOC relationships, and a generalized bonding model is required to explain the slopes of these SRs. We introduce such a model for the ZnO_*x*_H_*y*_ films and confirm that it also describes the behavior of reducible transition metal oxide films, such as TiO_*x*_H_*y*_, on metal substrates. We demonstrate how the SRs may be combined with grand canonical phase diagrams to predict film stability under conditions relevant to heterogeneous catalytic reactions, and we apply these insights to estimate which transition metals are likely to exhibit SMSI behavior under realistic environmental conditions. Finally, we discuss how SMSI overlayer formation for irreducible oxides, such as ZnO, is linked to hydroxylation and is mechanistically distinct from the overlayer formation for reducible oxides such as TiO_2_.

## Abstract 2

General principles of Strong Metal–Support Interactions (SMSI) overlayer formation have been elucidated using predictive models derived from ultrathin (hydroxy)oxide films on transition metal substrates.

## Graphical abstract

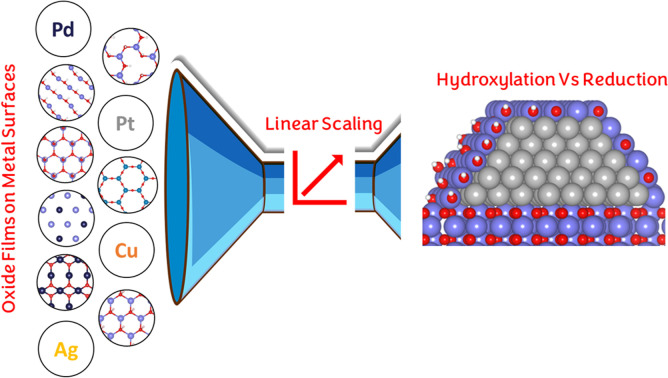

## Introduction

First principles-based density functional theory (DFT) calculations have long been employed to elucidate the fundamental principles of catalytic reactivity and to design improved catalytic materials. Linear scaling relationships (SRs), which correlate the binding energies of adsorbed reaction intermediates across a series of catalytic surfaces, have, in turn, emerged as central tools in these endeavors.^1^ Since the discovery of SRs on close packed and stepped metal surfaces,^2^ they have also been identified for other materials such as zeolites,^3^ alloys,^4^ metal oxides, sulfides, and nitrides.^5^

The physics behind SRs is generally explained using simple bond order conservation (BOC) principles. In particular, the BOC model for AH_*x*_ (A = O, H, C, N)-type adsorbates leads to the following expression (eqn (1)) for the slope of the SRs:^2^1
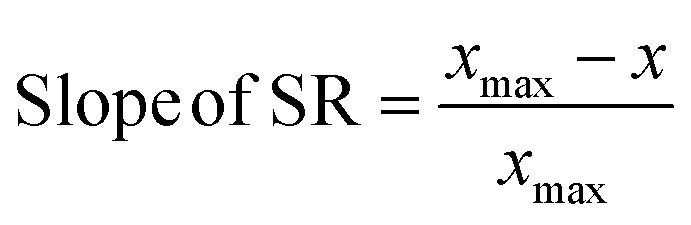
where *x*_max_ is the maximum valency of the central atom A and *x* is the number of H atoms binding to A. The BOC model works well for many materials, especially when the adsorbates interacting with the surface are p block elements and the bonding is covalent in nature. However, SRs have not found widespread application to the adsorption of more complex classes of materials and molecules on catalyst surfaces, and there is significant interest in extending SRs to such systems.

A particular class of materials that is common in heterogeneous catalysis, and for which SRs are beginning to be explored, is that of thin film transition metal oxides supported on metal substrates. Among other applications, these films play a central role in the celebrated Strong Metal–Support Interaction (SMSI), where oxide films partially cover metal nanoparticle surfaces. The studies of the SMSI date to the pioneering work of Tauster and colleagues, who discovered that hydrogen treatment could deactivate certain types of oxide-supported transition metal catalysts.^6^ In the intervening years, many additional studies have explored the origins of the SMSI and have described the phenomena in terms of the formation of an encapsulating oxide overlayer,^7–11^ as demonstrated by electron microscopy and related techniques.^12–15^ Furthermore, while it has traditionally been assumed that SMSI inhibits catalysis, recent studies have suggested that it can, in some cases, lead to improved catalytic properties.^16–18^ In spite of these advances, few broadly applicable principles exist to describe either the stability of ultrathin films on metal substrates, in particular, or the SMSI, in general.^19–21^ Also, most analyses, including atomic resolution single crystal STM studies^22^ and theoretical calculations, have been made on a system-specific basis. An important exception is the work of Plessow *et. al.*,^19^ who have recently demonstrated the existence of linear SRs for monolayer oxide films (V_2_O_3_, Ti_2_O_3_, TiO, and FeO) on the (111) surfaces of some transition metals. The authors reported that film formation energies scale linearly with the binding energies of single atoms of the corresponding transition metals (V, Ti, and Fe). Surprisingly, however, the SR slopes for some of the films deviated substantially from the BOC principle, strongly suggesting that new physics remains to be discovered for these systems. A similar phenomenon was observed by Choksi *et al.* for the SRs of *MOH and *MCH *vs.* *M (where M = Pt, Pd, Au)^23^ over transition metals, where inconsistent slopes with the BOC principle were also reported.

To rationalize the numerous observations concerning the behavior and structure of ultrathin films, comprising diverse structures, compositions, and substrates, we introduce a generalized class of SRs, applicable to both oxide and mixed hydroxy-oxide films, and propose a physicochemical explanation for the functional form of these SRs. We focus initially on zinc hydroxy-oxide films over transition metal surfaces, where the ZnO_*x*_H_*y*_/M (M = Ag, Au, Cu, Ir, Pd, Pt, Rh) system is selected because ZnO-supported nanoparticles play a major role in catalyzing industrially important reactions^24–26^ and are also known to form an SMSI like encapsulating layer under reaction conditions.^27^ An additional motivation for this choice of material is that bulk ZnO is an irreducible oxide, although its polar facets may show aspects of reducibility, such as hydroxylation or reconstruction.^28–30^ Therefore, its properties cannot be fully explained using the traditional hypothesis that the reduction of the support cation is necessary to exhibit SMSI, and the mechanistic driving force for encapsulation is not well understood. Furthermore, thin film ZnO has been characterized using ultra-high vacuum surface science techniques on Ag,^31^ Au,^32^ Pt,^33,34^ Cu,^35^ and Pd(111) (ref. 36) single crystals, permitting a robust comparison of our density functional theory (DFT) results with experiments. Finally, the formation of hydrogen-containing, ring-like Zn(OH)_5/6_ structures has been observed in reducing environments on certain metals such as Pd(111) (ref. 36) and Pt(111),^37^ while ZnO films on other metals, such as Ag(111), are less susceptible to hydroxylation, and no structural transformation has been observed under similar conditions.^38,39^

Motivated by the above considerations, we analyze ZnO, ZnOOH, Zn(OH)_5/6_, Zn(OH), and Zn(OH)_3/2_ films on Ag, Au, Cu, Ir, Pd, Pt and Rh(111) substrates. The specific stoichiometries are selected based on the experimental literature and our previous experimental/theoretical study of the ZnO/Pd(111) system.^40^ All of the structures exhibit classic SR behavior, which we interpret using a generalized bonding model. We then extend this model to other transition metal (hydroxy)oxide thin films and subsequently combine it with *ab initio* thermodynamic techniques to generate surface phase diagrams that provide insights into thin film behavior under high pressure conditions relevant to the SMSI. Finally, we extend our understanding beyond Zn-based films to the classic SMSI system containing reducible TiO_*x*_H_*y*_ films on metal substrates and compare their properties to those of the ZnO_*x*_H_*y*_ films. We confirm that the mechanistic origin of SMSI overlayer formation for reducible oxides such as TiO_2_ is linked to the reduction of the support cation to lower the oxidation state, while for irreducible oxides such as ZnO, the formation of hydroxylated films is the driving force for SMSI. The aggregate results illustrate the general principles of ultrathin film physics and SMSI behavior that may be useful in suggesting future heterogeneous catalysts that exploit SMSI-like phenomena.

## Methods

Periodic Density Functional Theory (DFT) calculations are performed using the Vienna *Ab Initio* Simulation Package (VASP).^41,42^ Projector Augmented Wave (PAW) potentials^43^ are used to model the core electrons. The generalized gradient approximation (GGA), in the form of the Perdew–Burke–Ernzerhof (PBE) functional, is employed,^44^ and the energy cutoff for the planewave basis set is taken as 500 eV. The Monkhorst–Pack *k* point scheme is used for all calculations. To ensure consistency in *k* point sampling over all unit cells, we ensure that the lattice constant times the number of *k* points is close to 30 Å in all cases. The (111) surfaces of metals Ag, Au, Cu, Ir, Pd, Pt, and Rh are modelled from converged face centered cubic (fcc) bulk structures with lattice constants of 4.15 Å, 4.16 Å, 3.63 Å, 3.87 Å, 3.94 Å, 3.97 Å, and 3.82 Å, respectively. The slabs consist of four layers with the bottom two layers constrained. The unit cells for the ZnO_*x*_H_*y*_ and TiO_*x*_H_*y*_ monolayer films are constructed using the procedure described in our previous study,^40^ wherein the interfacial strain in non-pseudomorphic, periodic overlayer structures is minimized^45^ (only structures containing less than 100 atoms in the substrate were considered). The film formation energy is calculated using eqn (2), where a metal slab, bulk ZnO, H_2_(g), and O_2_(g) are used as reference states:2
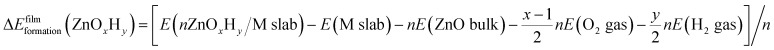


Bader charges are evaluated as described by Henkelman and coworkers,^46^ and Projected Crystal Orbital Hamilton Population (pCOHP) analysis is performed using the LOBSTER package.^47^

## Results and discussion

We begin by describing the SRs that are calculated for the ZnO_*x*_H_*y*_ films. Next, we introduce a generalized bonding model to rationalize the functional form and slopes of these SRs. We discuss how this formalism may be combined with pCOHP analysis to predict the slopes without the need for exhaustive DFT calculations, and we demonstrate that the approach can additionally be extended to films that contain transition metals other than Zn. We illustrate the utility of the results by combining the ZnO_*x*_H_*y*_ film SRs with *ab initio* thermodynamics to generate grand canonical surface phase diagrams that can, in turn, be used to estimate the likelihood of SMSI overlayer formation. Finally, we generalize these analyses to TiO_*x*_H_*y*_ films and discuss the chemical structure of both ZnO_*x*_H_*y*_ and TiO_*x*_H_*y*_ films under technical reaction conditions.

### Stability trends and scaling relationships in ZnO_*x*_H_*y*_ films

To probe the existence and nature of SRs in ZnO_*x*_H_*y*_ films, we evaluate the formation energies of ZnO, ZnOOH, Zn(OH)_5/6_, Zn(OH), and Zn(OH)_3/2_ films on Pt, Pd, Cu, Ag, Au, Ir, and Rh(111) substrates. In all cases, the strain minimized structures are linearly correlated with single atom adsorption energies, but the nature of these SRs varies considerably. As described further below, we broadly divide the SRs into three categories.

**Fig. 1:**
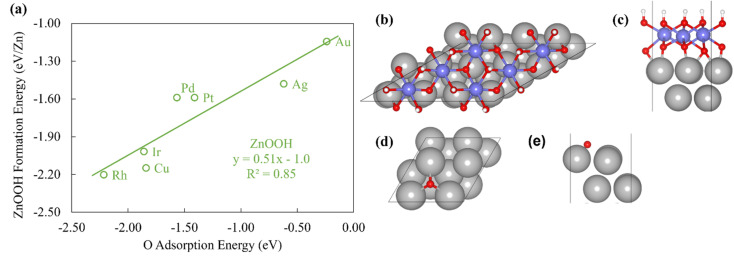
(a) The formation energy of ZnOOH films *vs.* the atomic oxygen adsorption energy. (b) Top view and (c) side view of ZnOOH/Pt(111). (d) Top view and (e) side view of O/Pt(111). The white, red, grey, and purple atoms are H, O, Pt and Zn atoms respectively.

The ZnOOH/M(111) films adopt a compact, space-filling structure on the fcc(111) surfaces. The films bind to the metal substrate through the O atom, and the film formation energies scale linearly with the O atom binding energy (Fig. 1a). Interestingly, this correlation holds in spite of the fact that the positions of the oxygen atoms with respect to the metal substrate atoms are different for each substrate, as the respective unit cells were varied to minimize the interfacial lattice strain. There is a close relationship between the scaling behavior of these structures and the traditional SRs for OH_*x*_ adsorbates observed by Abild-Pederson *et al.*^2^ (Fig. 1c). The number of bonds that the substrate makes with the film, (*x*_max_ − *x*), is calculated by assuming that the maximum valency of each element in the film is satisfied. As with OH_*x*_ species, the ZnOOH films bind exclusively through O and so *x*_max_, the maximum valency of an O atom, is equal to 2. Since O forms one bond with Zn, *x*_max_ − *x* should be equal to 1 to satisfy the maximum valency of O. The BOC principle thus predicts the scaling slope to be 0.5 (eqn (1)), consistent with the slopes determined from the full DFT calculations (Fig. 1a).

**Fig. 2:**
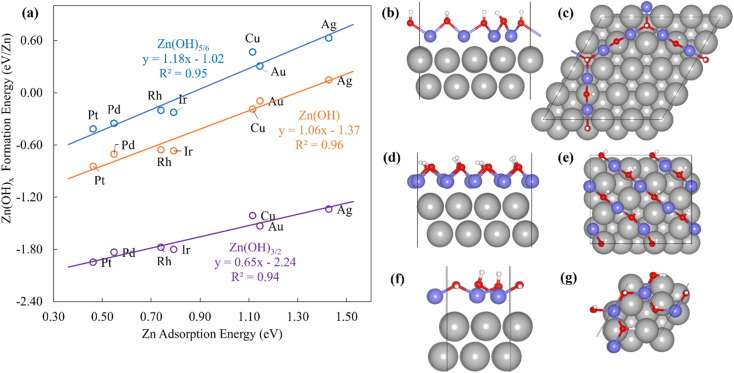
(a) Formation energies of Zn(OH)_*x*_ films on (111) transition metal surfaces *vs.* Zn atom adsorption energies. The blue, orange, and purple lines represent Zn(OH)_5/6_, Zn(OH), and Zn(OH)_3/2_ films, respectively. (b) Top and (c) side views of Zn(OH)_5/6_/Pt(111). (d) Top and (e) side views of Zn(OH)/Pt(111). (f) Top and (g) side views of Zn(OH)_3/2_/Pt(111).

**Fig. 3:**
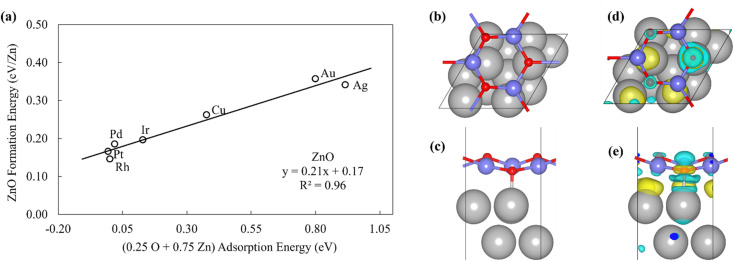
(a) The formation energy of ZnO films is plotted against linear combination of Zn atom and O atom adsorption energies. (b) Top view and (c) side view of ZnO/Pt(111). (d) Top view and (e) side view of the charge difference plot of ZnO/Pt(111). Yellow and light blue regions represent the accumulation and depletion of charge, respectively.^49^

The Zn(OH)_*x*_ (*x* = 5/6, 1, 3/2, where *x* is determined by the average number of OH per Zn in the unit cell) films adopt an open and linear structure on the fcc(111) surfaces (Fig. 2), resulting from the fact that each “OH” moiety in the film interacts with two adjacent Zn ions, where the Zn–OH coordination environment is the same for all the Zn atoms in a given film (we note, in passing, that these structures could have promising properties for catalytic applications since they do not space fill the entire metal surface and hence expose a combination of metallic and metal hydroxide sites). The films bind to the substrate exclusively through their Zn moieties, and the film formation energies scale linearly with the Zn atom binding energies (Fig. 2a). Assuming the maximum valency of Zn (*x*_max_) equal to 2, the BOC model predicts slopes equal to 0.58, 0.50 and 0.25 for Zn(OH)_5/6_, Zn(OH), and Zn(OH)_3/2_ films respectively. These values are substantially different from the calculated slopes of 1.18, 1.06, and 0.65. We analyze this unanticipated result below. The ZnO/M films have a space filling structure on the fcc(111) surfaces, with both O and Zn moieties interacting with the metal substrates. This multidentate adsorption is associated with a scaling relationship between the film formation energies and a linear combination of the binding energies of the corresponding transition metal atoms and oxygen (Fig. 3a). These observations are, in fact, reminiscent of the multidentate SRs proposed by Jones *et al.*^48^ for small molecules.3
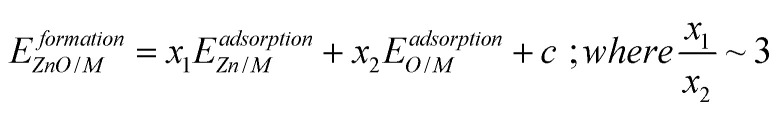


The inspection of the structures in Fig. 3 suggests that only a few of the O atoms in the film interact directly with the substrate. This phenomenon can also be clearly observed by the analysis of charge difference plots (Fig. 3d and e), which indicate that only one of the three oxygen atoms in a unit cell (on top of a Pt atom) forms a bond with the underlying Pt substrate. Similar structures are observed on other metal substrates, with the charge difference plots showing that only a fraction of oxygen atoms interact directly with the surface metal atoms. This result is likely due to the underlying hexagonal symmetry of the metal surface, which allows only 1/3 of the O atoms to be exactly on top of metal atoms. In contrast, all Zn atoms interact with the metal substrate, leading to the 3 : 1 ratio observed in the SR descriptor (eqn (3)). We note that it is difficult to rigorously relate these multidentate SRs to simple bond counting arguments since the 3 : 1 ratio is not, itself, determined by any formal bond counting principle, and in any case, the freestanding ZnO structures do not, themselves, follow simple bond counting principles for Zn and O.

Finally, we further note that the ZnO film formation is weakly endothermic. Thus, although such films might be observed at relatively low total surface coverages of Zn, at higher coverages and temperatures, bulk ZnO structures would be expected to nucleate and grow on the metal substrates.

### Rationalizing the slopes in the scaling relationships

As described above, the BOC model fails to accurately describe scaling relationships when the films interact with the metal surface *via* their Zn moieties. These limitations are related to the breakdown of an underlying assumption of the BOC model, which is that the strength of a single bond between the substrate and the central adsorbate atom is constant. This assumption can, in turn, be rationalized using effective medium theory (EMT), as described by F. Abild-Pedersen *et al.*^2,50^ and explained briefly here. For a molecule AH_*x*_ near a metal surface, the optimal electron density (*n*_o_) required by the central atom *A* is provided by both the surface (*n*_surf_) and the surrounding hydrogen atoms such that *n*_o_ = *n*_surf_ + *xn*_H_. For a closed-shell molecule, AH_*x*max_, all of the electron density must be provided by the hydrogen atoms, and so *n*_o_ = *x*_max_*n*_H_. Combining these expressions gives 
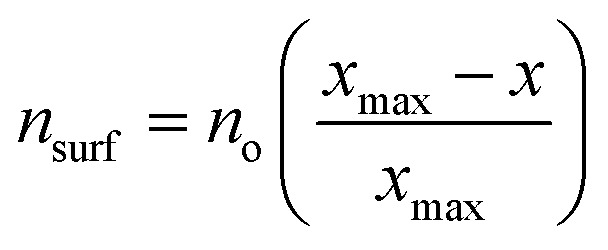
, and since *n*_surf_ is also proportional to the binding energy of the AH_*x*_ species, eqn (1) is recovered. This analysis holds provided that *n*_o_ is the same for A for any value of *x*. This is true for simple adsorbates such as O, H, C, and N because the oxidation state, and hence the local electron density, of these species is the same for all AH_*x*_-type adsorbates. However, it is not valid for Zn since its oxidation state changes in a complex manner among the various ZnO_*x*_H_*y*_/M(111) films.

To illustrate these effects, we analyze the charge gained by different metal substrates due to adsorption of Zn-containing thin films using Bader analysis (Table 1).^46^ This analysis is not intended as a quantitative, or even fully comprehensive, description of bonding between the films and the metal substrates, but it does serve to provide qualitative insights into the corresponding trends. The results indicate that the quantity of the charge transferred to the films, divided by the charge transferred to a single Zn atom adsorbed on the surface, is approximately constant across all substrates (Table S3†). Assuming that the charge transfer is in some way correlated with the corresponding binding energies, these results are consistent with the existence of linear relationships between the formation energies of the various films and the Zn atom adsorption energy. However, the magnitudes of these charge transfer ratios are not self-evidently consistent with EMT and BOC principles. For example, the ratio of Bader charges gained by substrates for Zn(OH)_5/6_ films to that for single Zn adsorption is on average equal to 0.93, while the ratio drops by only a factor 0.86, to 0.80, for Zn(OH) films. In the former cases, OH moieties are bonded to either two or three Zn atoms, and in the latter, each OH is bonded to two Zn atoms. Given these qualitatively significant differences in bonding environments, one might in turn expect a more significant change in the charge transfer ratios. For comparison, in the traditional SR for *O *vs.* *OH binding energies, the addition of H to *O leads to a much more significant reduction in the Bader charge lost by the substrate, by a factor of 0.62. These comparisons provide further evidence that the assumptions of traditional BOC arguments do not hold for the adsorption of (hydroxy)oxide films on metal surfaces.

**Table 1:** Bader charge on the substrate due to the ZnO_*x*_H_*y*_ ultrathin films. The negative sign indicates that the substrate gains electrons

	Bader charge on the substrate per Zn (e^−^)						
	Ag	Au	Cu	Ir	Pd	Pt	Rh
Zn	−0.17	−0.37	−0.14	−0.36	−0.33	−0.46	−0.27
Zn(OH)_5/6_	−0.17	−0.36	−0.13	−0.33	−0.31	−0.40	−0.25
Zn(OH)	−0.14	−0.29	−0.14	−0.28	−0.25	−0.32	−0.21
Zn(OH)_3/2_	−0.08	−0.19	−0.07	−0.15	−0.16	−0.19	−0.11

The apparent violation of the BOC principles can be explained by relaxing the BOC model assumption that *n*_o_ is constant and assuming that *n*_o_ varies from one film stoichiometry to another. Effectively, the Zn–metal surface(M) bond strength and the Zn oxidation state are not constant for all film structures.

To account for the fact that the single Zn–M bond strength varies for different Zn-containing films, we propose the following generalized SR (see the ESI† for a derivation):4
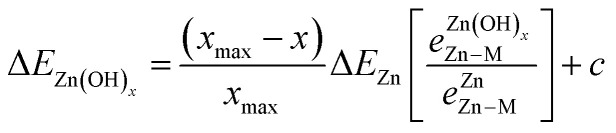
where *e*^Zn^_Zn–M_ is the bond strength between Zn and M for a single adsorbed Zn atom, and 
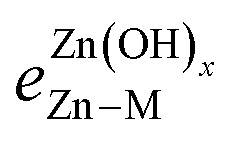
 is the bond strength between Zn and M for adsorbed Zn(OH)_*x*_ species. In the traditional SRs, the bond strength terms are constant and cancel one another, but when the bond strength varies, the terms change the slope of the SR. Motivated by this expression, we can draw a few general conclusions about the adsorption of the Zn(OH)_*x*_ films considered in this study. Firstly, since we observe a linear SR for Zn(OH)_*x*_ films, we infer that the bond strength ratios 
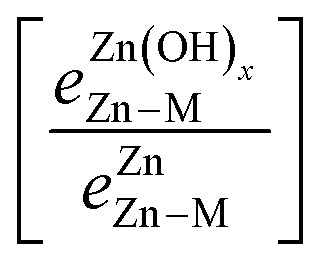
 must be constant across metal substrates but not equal to one. Secondly, we can back calculate the bond strength ratios by using the slopes determined from DFT calculations. Using this strategy, we observe that the value of bond strength ratios is 2.02, 2.12 and 2.60 for Zn(OH)_5/6_, Zn(OH), and Zn(OH)_3/2_, respectively. The ratios clearly vary depending on the bonding environment, and there is no obvious trend to these values. Therefore, the generalized SR (eqn (4)) is not truly predictive unless an independent procedure to estimate the bond strength ratios can be identified.

A promising approach to enhance the predictive power of the modified scaling analysis is to estimate bond strength ratios using Projected Crystal Orbital Hamilton Population (pCOHP) analysis. Integrating over pCOHP up to the Fermi level (so-called “ICOHP” values, where “I” denotes integration) gives an approximate bond strength value. Therefore, the ICOHP values can facilitate independent estimation of the bond strength ratio 
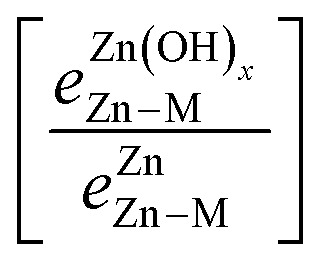
 in eqn (4). These values are normalized by the number of bonds between Zn and M to obtain a single Zn–M bond strength for fair comparison between the disparate films. As stated earlier, the number of Zn–M bonds is estimated by assuming that all the elements in the film satisfy their maximum valency, which is 2, 2, and 1 for Zn, O, and H, respectively.

We find that the normalized ICOHP values for Zn–M interactions in Zn(OH)_5/6_ films are approximately twice the values for similar interactions in single Zn atom adsorption, when M = Ir, Pt, Pd and Rh (Table S4†), which is consistent with our predictions from the scaling slopes. The agreement is less quantitative, however, for the coinage metals (Ag, Au, and Cu), which is most likely due to the complete filling of the d-bands in these elements. Similarly, for ZnOH films, the ICOHP values are slightly higher than for the Zn(OH)_5/6_ films, and the Zn(OH)_3/2_ films have higher values than both ZnOH and Zn(OH)_5/6_, again consistent with the results from the scaling slopes. In the next section, we generalize these observations and show that adding more films can further improve the predictive power of these correlations.

### Extension to other transition metal films

To assess the generality of eqn (4), we have extended the analysis to two additional classes of transition metal hydroxy(oxide) films that have been analyzed in the literature.^19,23^ The first system we consider is the scaling of *PtOH *vs.* *Pt, where Pt is, in this case, a single adatom adsorbed on various transition metal substrates. Choksi and colleagues reported that, for three-coordinated Pt adsorbates, the slope of the SR is equal to 1.18, which differs from the predictions of the standard BOC model. Our analysis is consistent with these results (Fig. S1†), and Bader analysis (Table S5†) reveals that Pt gains charge from the metal substrates. However, the charge lost by the substrate is nearly identical in the *Pt and *Pt(OH) cases. This result is similar to that of the Zn(OH)_*x*_ films, where there was little change in the charge gained by the substrates between *Zn adatoms and *Zn(OH)_5/6_ films, and again suggests that significant deviations exist from the BOC scaling models. These deviations for *PtOH can, in turn, be readily explained using eqn (4), with the Pt–M bond strength increasing from *Pt to *PtOH. We discuss this result in more detail below.

The second system that we consider is the scaling of *TiO_3/2_*vs.* *Ti on fcc(111) metal substrates. The calculated slope is equal to 0.25, which is similar to what was observed by Plessow *et al.*^19^ (Fig. S2†). If we assume that the maximum valency for Ti and O is equal to 4 and 2 respectively, the number of bonds between Ti and the substrate is equal to 1. Using eqn (1), we predict that the BOC scaling slope will equal 0.25, which agrees with our DFT results. Furthermore, Bader charge analysis (Table S6†) reveals that the metal substrates in the TiO_3/2_ films on average gain charge equal to 0.21 times the charge gained for a single Ti adsorbate, which is close to the BOC prediction of 0.25. In addition, the Bader charge on Ti itself is not significantly affected, suggesting that the bond order of Ti is similar in both situations. We also considered the scaling of *TiO *vs.* *Ti (Fig. S3†) and *TiOH *vs.* *Ti (Fig. S4†). Like *TiO_3/2_, these films bind through Ti to the substrate. The scaling slopes deviate from BOC principles, as with the Zn(OH)_*x*_ films. However, unlike Zn(OH)_*x*_ films, BOC overpredicts the slope for both TiO and TiOH. According to BOC, the slopes for TiO and TiOH should be 0.50 and 0.75 respectively, which are higher than the calculated slopes in the SRs.

**Fig. 4:**
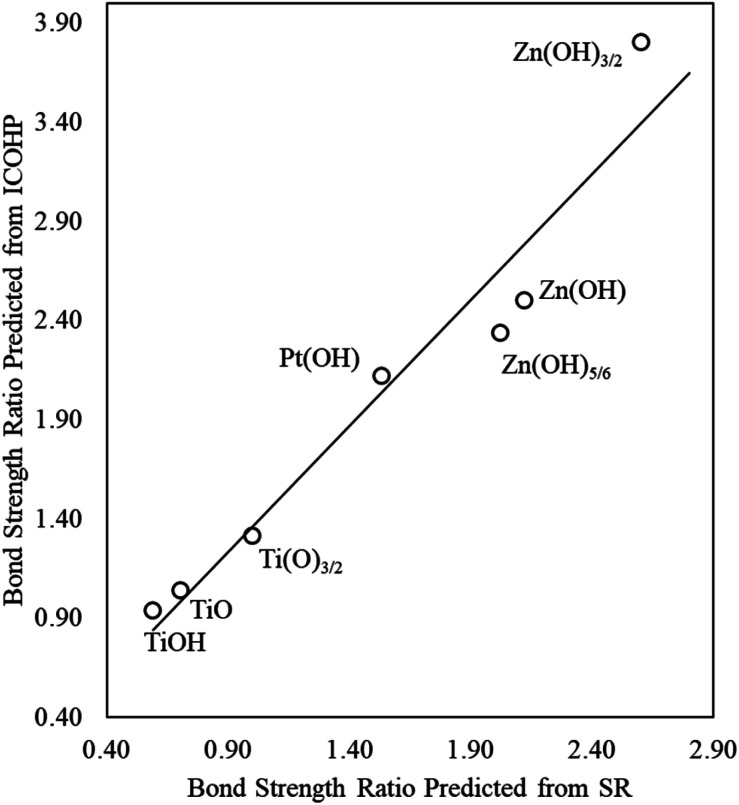
The bond strength ratio of film to metal atom adsorption calculated directly from ICOHP analysis (vertical axis), averaged over all transition metal substrates considered in this study, and estimated from DFT-calculated SR slopes (horizontal axis).

We again use ICOHP analysis to estimate the bond strength ratios (eqn (4)) for both Pt- and Ti-containing ad-species and compare them to values predicted from the SR slopes. As seen in Fig. 4, for a given substrate, the bond strength ratios calculated *via* ICOHP analysis and averaged over the metal substrates approximately scale with the bond strength ratios extracted from the scaling slopes. Remarkably, the relationship holds for both reducible (Pt and Ti) and irreducible oxides (Zn). This relationship, therefore, provides a starting point for estimating the scaling relationships for any type of thin film that binds to fcc(111) metal substrates through the film's metal moieties. Although the parameters in the scaling relationships may depend, to some extent, on the particular functional used (Plessow *et. al.*^19^ have reported that the slopes of certain SRs vary with the functional), we expect that the explanatory power of generalized scaling theory will be preserved for any given functional.

### Application of scaling relationships and comparison to surface science experiments

**Fig. 5:**
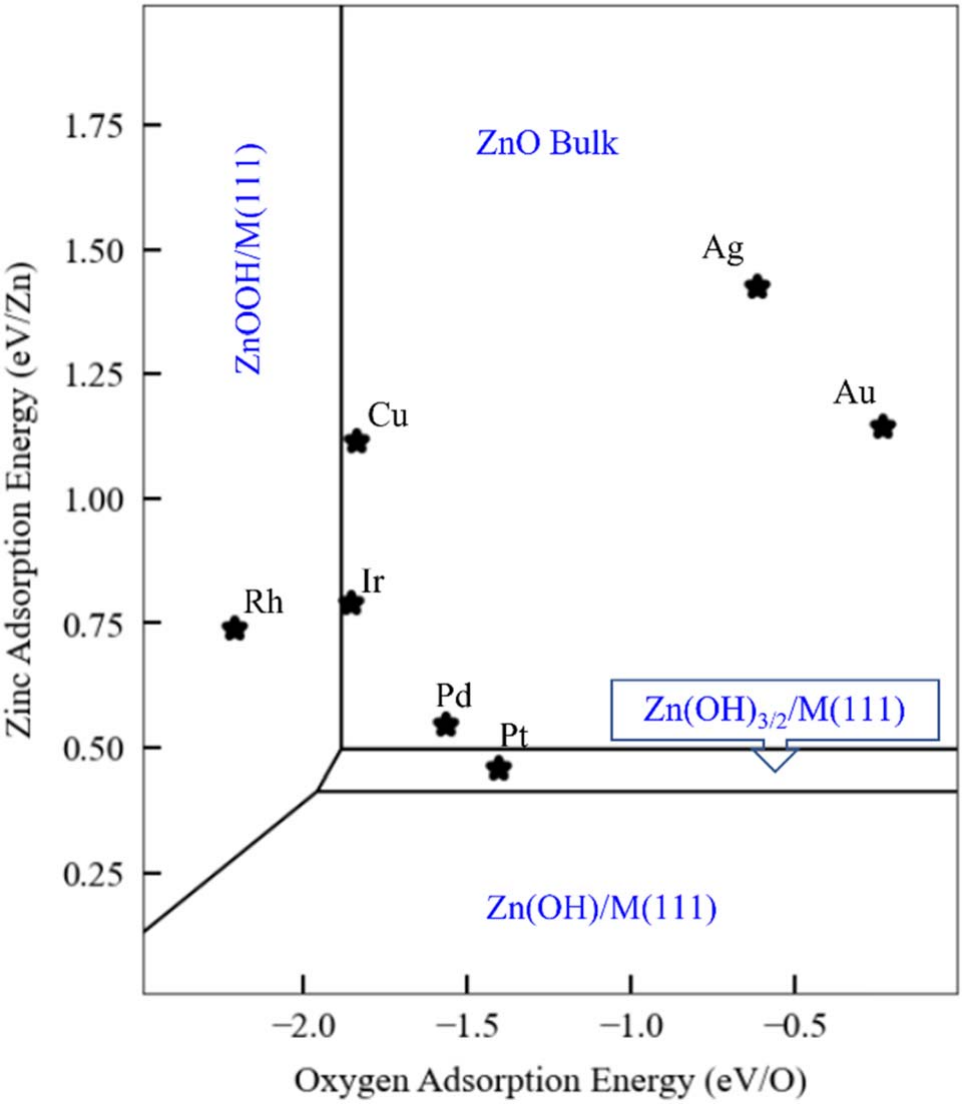
Phase plots for ZnO_*x*_H_*y*_ films on transition metal substrates with H_2_ (g) and O_2_(g) reservoirs at 10^−7^ mbar and 550 K, and with bulk ZnO as a solid-state reservoir. The oxygen binding energy and zinc binding energy are the independent descriptors. Scaling relationships described in the text are used to estimate the relevant film formation energies. The most stable structures for the given descriptor values are marked in blue. The black lines indicate the phase boundaries.

As discussed in the Introduction, ZnO_*x*_H_*y*_ monolayers on metal substrates, including Ag,^31^ Au,^32^ Pt,^33^ Cu,^35^ and Pd(111),^36^ are among the most widely studied ultrathin film inverse catalyst models. To illustrate how scaling relationships can facilitate the computational study of such systems, we use the SRs developed in the previous sections to generate a phase map for ZnO_*x*_H_*y*_ adsorption on the (111) facets of fcc transition metals (Fig. 5) at fixed values of the H, O, and Zn chemical potentials (grand canonical formalism). We include all ZnO_*x*_H_*y*_ structures considered in this study, as well as metal–Zn surface alloys with a 0.25 coverage of Zn (the formation energy of these alloys follows a SR through the Zn binding energy – Fig. S5†). Since the SRs imply that the formation energies of ZnO_*x*_H_*y*_ films on (111) surfaces can be accurately calculated using just the O and Zn binding energies, we choose these descriptors as our axes in the phase diagram. To facilitate comparison with surface science experiments, the hydrogen chemical potential is fixed at the corresponding H_2_ gas potential at 10^−7^ mbar and 550 K. Gao *et al.* have discussed the challenges of assigning exact chemical potentials to O and Zn in similar systems.^40^ As a first approximation, we assume that the Zn chemical potential is determined by bulk ZnO, and the O chemical potential is fixed at the corresponding O_2_ gas potential at 10^−7^ mbar and 550 K. The surface free energies are determined at each value of the O and Zn binding energies by calculating the film formation energies from the scaling relationships and using the values of the chemical potentials provided by Gao *et al.* We note that these chemical potentials are not always easy to determine for given experimental conditions, and metastable structures may even be observed at lower temperatures due to kinetic limitations.

Fig. 5 presents a general phase map that is, in principle, applicable to any fcc(111) metal or alloy surface. For reference, we have marked the positions of several common transition metals. Remarkably, the analysis accurately predicts that the Pt(111) surface can form ZnO_*x*_H_*y*_ species under UHV conditions, as observed by experiments.^38^ The stabilization of these structures is attributed to the strong binding of Zn to the Pt(111) surface. In contrast, on Ag(111) and Au(111), bulk ZnO formation is favored due to the weak interactions between these metals and Zn. On Pd(111), the zinc hydroxy structures are metastable when referenced to bulk ZnO, as the Zn binding energy is slightly weaker than on Pt. These observations are also consistent with experiments,^40^ as Gao *et al.* observed that Zn(OH)_*x*_ films on Pd(111) are metastable under many conditions. On Cu(111), Mahapatra *et al.*^51^ observed that, when depositing ZnO/Cu(111), the surface Cu can be oxidized to form CuO_*x*_. Although we have not explicitly considered copper oxidation in our study, this result is broadly consistent with the prediction (Fig. 5) that Cu falls in the ZnO monolayer region. However, we note that Cu is very close to the ZnOOH film boundary, and ZnOOH films might form at higher H chemical potentials. To our knowledge, there are no surface science experiments on Ir(111) and Rh(111) to which to make meaningful comparisons.

Finally, as briefly noted above, we emphasize that the particular ZnO_*x*_H_*y*_ species that form, and the locations of the phase boundaries in the phase plot, will vary according to the O, H, and Zn chemical potentials. Using these principles, we can further extend our UHV analyses to more realistic reaction conditions. As an example, we fix the hydrogen and oxygen chemical potentials to those of the corresponding gaseous species at 1 bar and 550 K. Under these conditions, the phase plot analysis (Fig. S7†) predicts that all metal substrates should stabilize Zn(OH)_*x*_ films, with a likely stoichiometry of Zn(OH)_3/2_. A complementary perspective is given in Fig. S8† for ZnO_*x*_H_*y*_/Pt(111), where the two axes correspond to hydrogen and oxygen chemical potentials, and hydrogen-containing phases are again seen to be favored for a significant range of these chemical potential values. A number of studies have indeed reported the existence of hydroxylated Zn species on Cu(111) under methanol synthesis reaction conditions,^25^ which is consistent with these predictions. We note, in passing, that these Zn(OH)_*x*_ structures have an open geometry that also exposes bare metal sites. As such, a multifunctional site distribution, comprising both metal and Zn(OH)_*x*_ sites, is present, leading to potentially exciting catalytic properties for these types of thin films and for SMSI catalysis more generally.

The generalized thermodynamic scheme described above is not confined to ZnO_*x*_H_*y*_ films and can also be employed to systematically study other films, including reducible oxides. To illustrate this point, we return to the example of TiO_*x*_H_*y*_ films and analyze their stability under SMSI conditions. As described earlier, the TiO_3/2_, TiO, and TiOH films follow linear scaling relationships, and their formation energy can be described using only the Ti adsorption energy. The SRs are thus independent of oxygen binding energy, and a phase diagram similar to Fig. 5 for Ti would have only a single dimension. Rather than presenting the diagrams in this form, however, we choose to replace the *x*-axis with the oxygen chemical potential. This approach allows us to describe phase transition between the various Ti bulk oxides, whose stability depends on the oxygen chemical potential. In practical terms, these different chemical potential states could be accessed using a procedure similar to that described by Zhang *et al.*^52^ who have reported that hydrogen in the Pd/TiO_2_ SMSI system controls the oxygen chemical potential through equilibrium with water (2H_2_ + O_2_ → 2H_2_O) which, in turn, determines overlayer film stability.

**Fig. 6:**
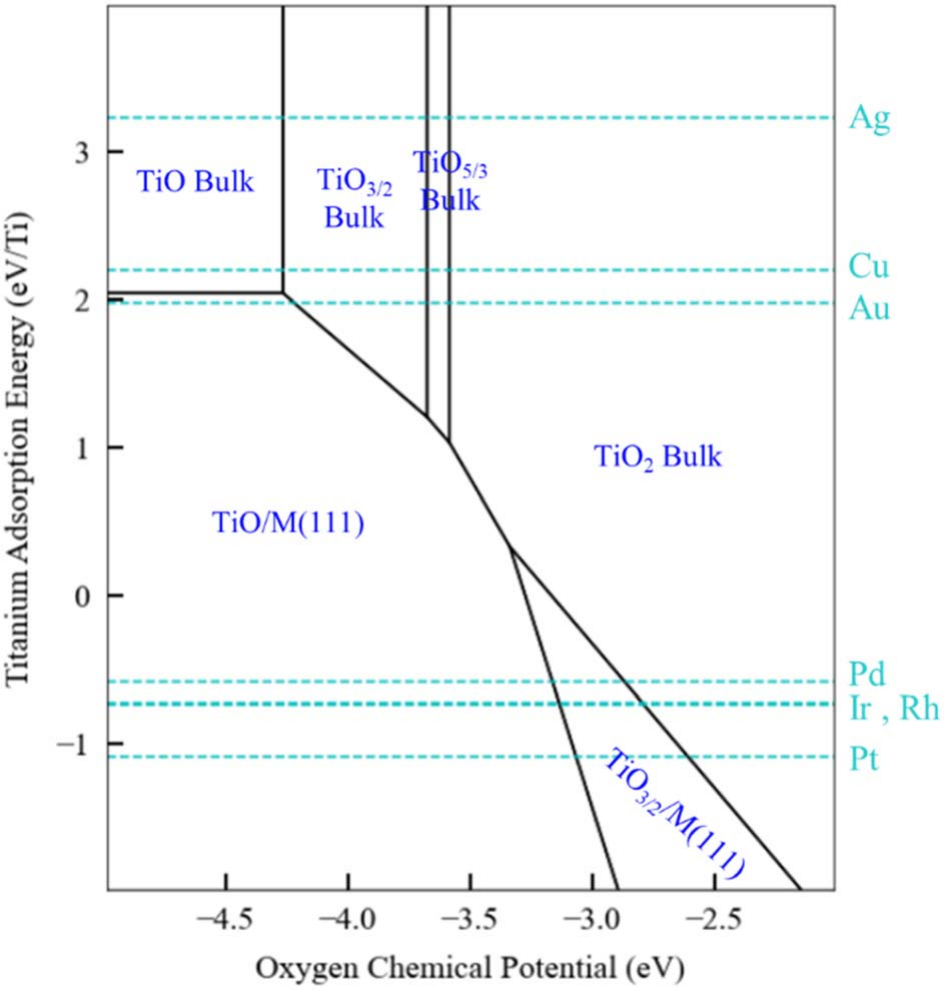
Phase plots for TiO_*x*_H_*y*_ films on transition metal substrates with a H_2_ (g) reservoir at 1 bar and 550 K, and with bulk Ti as a solid-state reservoir. The oxygen chemical potential (eV) and Ti binding energy are the independent descriptors. Scaling relationships described in the text are used to estimate the relevant film formation energies. The most stable structures for the given descriptor values are marked in blue. The black lines indicate the phase boundaries. The light blue dotted lines specify the Ti binding energy for a specific metal.

The phase map presented in Fig. 6 illustrates these concepts. Bulk TiO_2_ is the most stable oxide phase, and very low oxygen chemical potentials are required to reduce it. However, the TiO and Ti_2_O_3_ films can be stabilized on Pd, Ir, Rh, and Pt substrates, and therefore the onset of reduction of Ti to form these films occurs at a higher oxygen chemical potential than the corresponding bulk process. This suggests that SMSI could be possible when these metals are supported by TiO_2_, and SMSI has, indeed, been experimentally demonstrated for these systems.^7,52^ On the other hand, the coinage metals do not interact strongly with the oxide films, and as seen in Fig. 6, the reduction of TiO_2_ on these metals does not lead to supported film formation (only bulk oxides are formed). The hydroxylated TiOH film is also considered in our analysis, and it is found to be unstable, even under conditions of high hydrogen chemical potential (1 bar and 550 K). Based on the available data, we therefore predict that the overlayer formation in Ti is not related to hydroxylation, as is the case for SMSI on ZnO. Rather, SMSI is simply linked to the reduction of TiO_2_.

We conclude by summarizing the implications of our results for the mechanistic origins of SMSI. Our results support the traditional explanation for SMSI on reducible oxide substrates such as TiO_2_, where SMSI overlayer formation is driven by the reduction of the cation and subsequent migration onto the metal surface. In contrast, SMSI on irreducible bulk ZnO cannot be explained using the same mechanism. In that case, hydroxylation plays a key role in stabilizing ZnO_*x*_H_*y*_ films and driving migration on the metal surfaces. These disparate mechanisms illustrate the rich physics and chemistry of SMSI and may, ultimately, point to strategies to suppress or modify SMSI for desired applications.

## Conclusions

Although SRs in heterogeneous catalysis have been studied for more than a decade, the molecular-level reasons that such SRs deviate from the classic bond order conservation (BOC) principles are not fully understood. In the context of ultrathin (hydroxy)oxide metal films on transition metal substrates, which serve as important model systems for well-known catalytic phenomena such as the Strong Metal–Support Interaction, SRs have been observed but do not follow BOC principles. To explain these puzzling deviations, and to provide more predictive power for the study of ultrathin films and SMSI phenomena, we present a generalized SR, based on the assumption that film-surface binding may vary due to changes in the oxidation state, that explains the molecular-level interactions with the metal surfaces that are responsible for the breakdown of the BOC approximation. We demonstrate the explanatory and predictive power of these relationships for several common ultrathin film systems, and we further discuss how the SRs can lead to the identification of easier-to-calculate descriptors to estimate the film formation energies of complex thin film oxides over metal substrates. As an example, using just the Zn and O adsorption energies, it is possible to determine a comprehensive phase map for ZnO_*x*_H_*y*_ film adsorption on disparate transition metal surfaces at given H, O, and Zn chemical potentials. This map, in turn, suggests that hydroxylated films will form under technical reaction conditions. Therefore, the driving force for SMSI overlayer formation in ZnO is attributed to hydroxylation, as opposed to cation reduction, which is not possible for Zn. Contrasting these results with TiO_2_, where cation reduction does indeed drive SMSI film formation, reveals that SMSI can be caused by different mechanisms, and detailed thermodynamic analysis is necessary to show which metal surfaces are likely to promote SMSI under given environmental conditions, as well as the molecular structure of these films. These principles may, ultimately, open up the possibility of engineering new catalytically active sites through rational exploitation of SMSI-related phenomena.

## Data availability

The raw DFT data of this study are available on request from the corresponding author, J. P. G. The lattice matching algorithm can be found at https://github.itap.purdue.edu/GreeleyGroup/fga.

## Author contributions

K. J. S carried out the calculations and formal analysis with the guidance of Z. Z and J. P. G. The manuscript was written and edited by all authors. All authors have given approval to the final version of the manuscript.

## Conflicts of interest

There are no conflicts to declare.

## Supplementary Material

SC-014-D2SC06656D-s001
