# Supplementary material for: Universal properties of metal-supported oxide films from linear scaling relationships: elucidation of mechanistic origins of strong metal–support interactions
Source: Chem Sci. 2023 Feb 4;14(12):3206–14. doi: 10.1039/d2sc06656d (PMC10034000; doi:10.1039/d2sc06656d)
Supplement: SC-014-D2SC06656D-s001 [file SC-014-D2SC06656D-s001.pdf]

Electronic Supplementary Material (ESI) for Chemical Science.
This journal is © The Royal Society of Chemistry 2023

Universal properties of metal-supported oxide films from linear scaling relationships: Elucidation of mechanistic origins of strong metal-support interactions

*Kaustubh J. Sawant[1], Zhenhua Zeng[1], Jeffrey P. Greeley[1]**

[1]Charles D. Davidson School of Chemical Engineering, Purdue University

*Corresponding author e-mail: jgreeley@purdue.edu

S1. Bonding theories

S1.1. Traditional scaling relationships

$\Delta E_{AHx} = (x_{max} - x)e_{A-M} + c_1$ (S.1)

$\Delta E_{A} = (x_{max})e_{A-M} + c_2$ (S.2)

Divide 1.1 by 1.2 to obtain

$$\Delta E_{AHx} = \frac{(x_{max} - x)}{x_{max}}\Delta E_A + c_3 \quad \text{(S.3)}$$

where

$x_{max} =$ maximum valency of *A*,

$e_{A-M} =$ average bond strength between *A* and Zn,

$\Delta E_x =$ adsorption energy.

Assumption : e_{A-M} is constant between equation 1 and 2 and therefore cancels.

S1.2. Metal-Metal bond scaling

$\Delta E_{Zn(OH)x} = (x_{max} - x)e^{1}_{Zn-M} + c_1$ (Covalent bonding) (S.4)

$\Delta E_{Zn} = (x_{max})e^{2}_{Zn-M} + c_2$ (Metallic bonding) (S.5)

Divide 1.4 by 1.5 to obtain

$$\therefore \Delta E_{Zn(OH)_x} = \frac{(x_{max} - x)}{x_{max}} \Delta E_{Zn} \left[\frac{e_{Zn-M}^{Zn(OH)}}{e_{Zn-M}^{Zn}} \right] + c \qquad \text{(S.6)}$$

S2. DFT energy equations

$$\Delta E_{Zn_x/M}^{alloy\,formation} = \left[E_{Zn_x/M} - E_{M\,slab} - xE_{Zn\,bulk} + xE_{M\,bulk} \right] / x \qquad \text{(S.7)}$$

$$\Delta E_{N/M}^{Adsorption} = \left[E_{N/M} - E_{M\,slab} - E_{N\,bulk} \right] \qquad \text{(S.8)}$$

where N = (Zn, Pt, Ti, O) and M = (Ag, Au, Cu, Ir, Pd, Pt, Rh)

$$\Omega = x_1 \Delta E_{Zn} + x_2 \Delta E_O + c \qquad \text{(S.9)}$$

where Ω= free energy of given surface phase

x_1 and x_2 are slopes of scaling relationships build using the right reference for bulk *ZnO*, H_2(g) and O_2(g).

Table S1 : Bader charge gained by the OH attached to the Zn in *Zn(OH)*$_x$ films and normalized by number of OH in the films.

	Bader charge on OH per Zn (e^-)						
	Ag	Au	Cu	Ir	Pd	Pt	Rh
$Zn(OH)_{5/6}$	-0.57	-0.56	-0.56	-0.55	-0.55	-0.55	-0.56
Zn(OH)	-0.66	-0.66	-0.66	-0.63	-0.64	-0.65	-0.65
$Zn(OH)_{3/2}$	-0.98	-0.97	-0.97	-0.96	-0.95	-0.97	-0.96

Table S2 : Bader charge gained by the Zn per N_{Zn} in *Zn(OH)*$_x$ films.

	Bader charge on Zn (e^-)						
	Ag	Au	Cu	Ir	Pd	Pt	Rh
Zn	0.17	0.37	0.15	0.36	0.33	0.46	0.27
$Zn(OH)_{5/6}$	0.74	0.91	0.69	0.88	0.86	0.95	0.81
Zn(OH)	0.79	0.95	0.81	0.91	0.89	0.97	0.86
$Zn(OH)_{3/2}$	1.07	1.16	1.04	1.11	1.10	1.16	1.07

Table S3 : Ratio of Bader charge on metal substrate for *Zn(OH)*$_x$ films with respect to the Bader charge on metal surface for single Zn adsorption.

	Ag	Au	Cu	Ir	Pd	Pt	Rh
$Zn(OH)_{5/6}$	1.02	0.96	0.91	0.91	0.93	0.87	0.95
Zn(OH)	0.80	0.80	0.99	0.76	0.75	0.71	0.79
$Zn(OH)_{3/2}$	0.47	0.51	0.48	0.42	0.47	0.42	0.40

Table S4 : Average ICOHP values for Zn-metal interactions.

	**ICOHP values for Zn-M interaction**						
	Ag	Au	Cu	Ir	Pd	Pt	Rh
Zn-M interaction for Zn atom(divided by 2)	-0.37	-0.47	-0.39	-0.72	-0.74	-0.73	-0.74
Zn-M interaction for $Zn(OH)_{5/6}$(divide by 7/6)	-1.07	-1.20	-0.99	-1.57	-1.52	-1.48	-1.58
**Ratio of Zn-M interaction in $Zn(OH)_{5/6}$ vs Zn atom**	**2.91**	**2.54**	**2.53**	**2.17**	**2.05**	**2.03**	**2.14**
Zn-M interaction for Zn(OH)(divide by 1)	-1.07	-1.20	-0.99	-1.57	-1.52	-1.48	-1.58
**Ratio of Zn-M interaction in Zn(OH) vs Zn atom**	**3.02**	**2.72**	**2.85**	**2.27**	**2.15**	**2.16**	**2.32**
Zn-M interaction for $Zn(OH)_{3/2}$ (divide by 0.5)	-1.74	-1.58	-1.67	-2.79	-2.54	-2.46	-2.71
**Ratio of Zn-M interaction in $Zn(OH)_{3/2}$ vs Zn atom**	**4.73**	**3.35**	**4.25**	**3.85**	**3.43**	**3.36**	**3.66**

Table S5: Bader charges on substrates after adsorption of *Pt and **Pt(OH)*.

	**Bader charge gained by substrate per Pt (e^-)**						
	Ag	Au	Cu	Ir	Pd	Pt	Rh
Pt	0.36	0.11	0.43	0.20	0.18	0.07	0.27
Pt(OH)	0.33	0.10	0.41	0.19	0.14	0.05	0.25

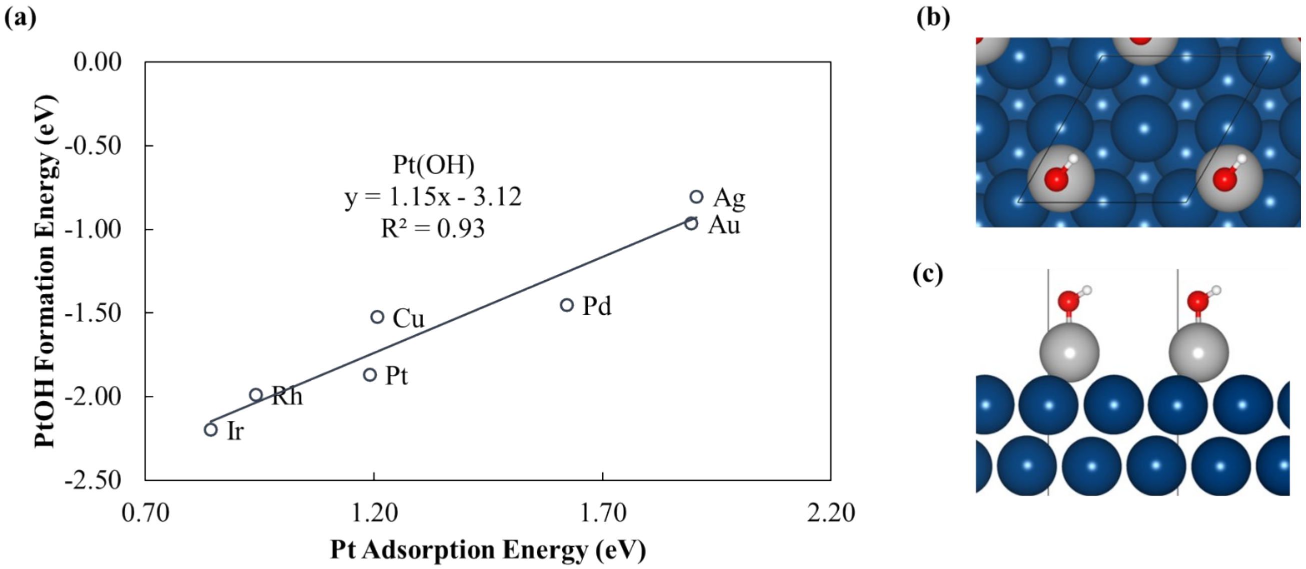

Figure S1 : (a) Scaling relationship between **PtOH* adsorbates (0.25 coverage) and Pt single atom adsorption energy. (b) Side and (c) top view of *PtOH*/Pd(111) with 0.25 coverage.

Table S6: Bader charge on the substrate after adsorption of *Ti and *Ti_2O_3

	Bader charge gained by substrate per Ti (e^-)						
	Ag	Au	Cu	Ir	Pd	Pt	Rh
Ti	-2.79	-2.40	-2.67	-2.17	-2.17	-2.09	-2.20
Ti_2O_3	-0.36	-0.50	-0.47	-0.51	-0.56	-0.59	-0.42

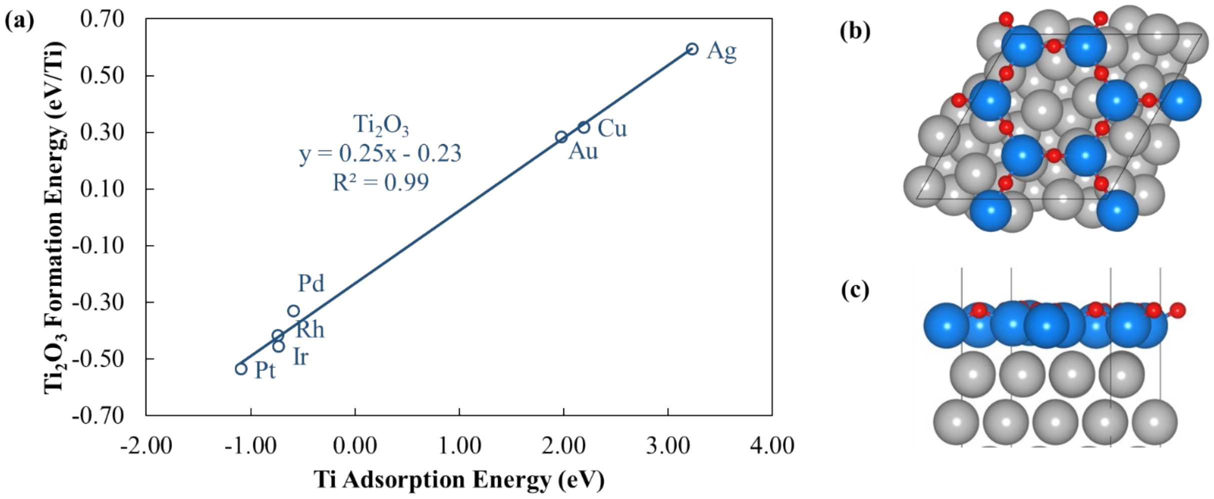

Figure S2 : (a) Scaling relationship between *Ti_2O_3*/M(111) formation energy and Ti single atom adsorption energy. (b) Top and (c) side views of *Ti_2O_3*/Pt(111) film. Light blue atoms correspond to Ti atoms.

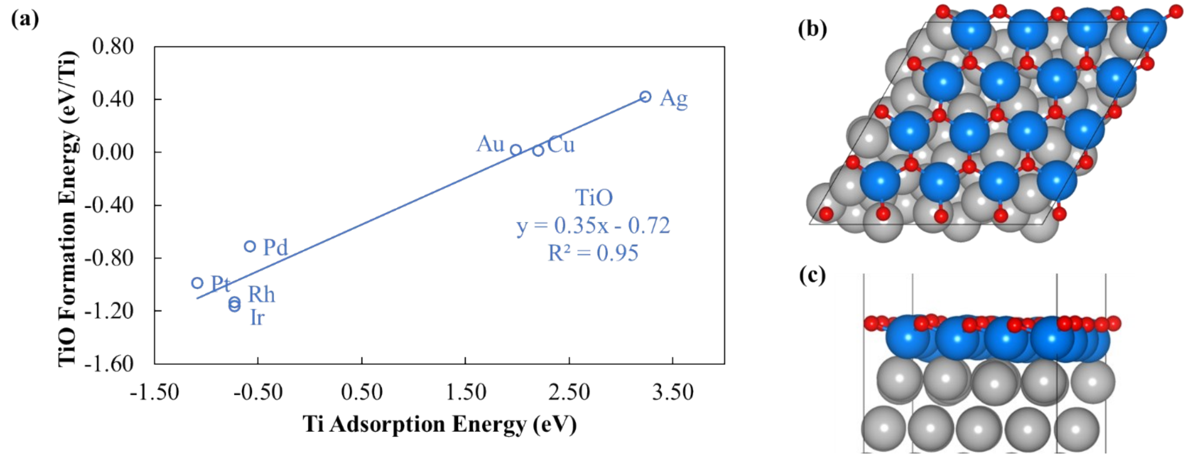

Figure S3 : (a) Scaling relationship between *TiO*/M(111) formation energy and Ti single atom adsorption energy. (b) Top and (c) side views of *TiO*/Pt(111) film.

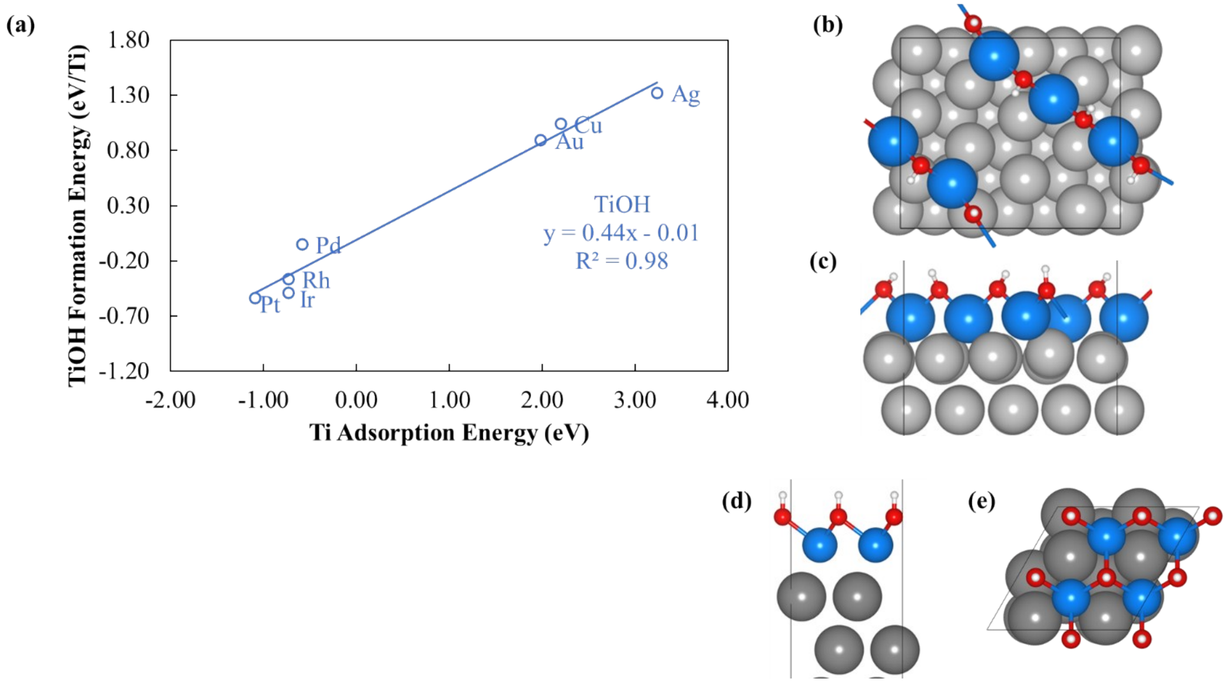

Figure S4 : (a) Scaling relationship between TiOH/M(111) formation energy and Ti single atom adsorption energy. (b) Top and (c) side views of TiOH/Pt(111) film. (d) Side and (e) top views of TiOH/Ag(111) film. Black atoms correspond to Ag. Please note that the TiOH films can be 1d or 2d depending on the underlying substrate. The most stable film for Pd, Pt, Rh, and Ir is 1d films, while for Ag, Au, and Cu it's 2d films. Figure S4b and c are good representative images of 1d films, while S4d and e are good representative images of 2d films. We used the most stable structure to make the scaling relationship in Figure S4a.

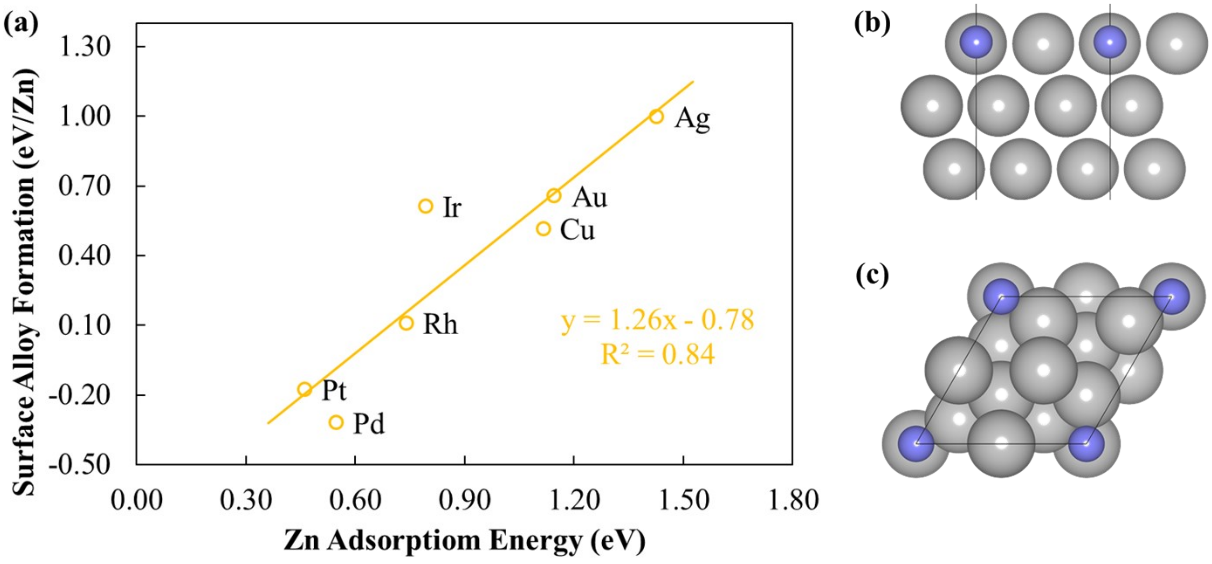

Figure S5 : (a) Scaling relationship between surface alloy formation energy (Equation S7) of Zn-M species (0.25 coverage) and Zn single atom adsorption energy. (b) Side and (c) top view of PtZn surface alloy with 0.25 coverage.

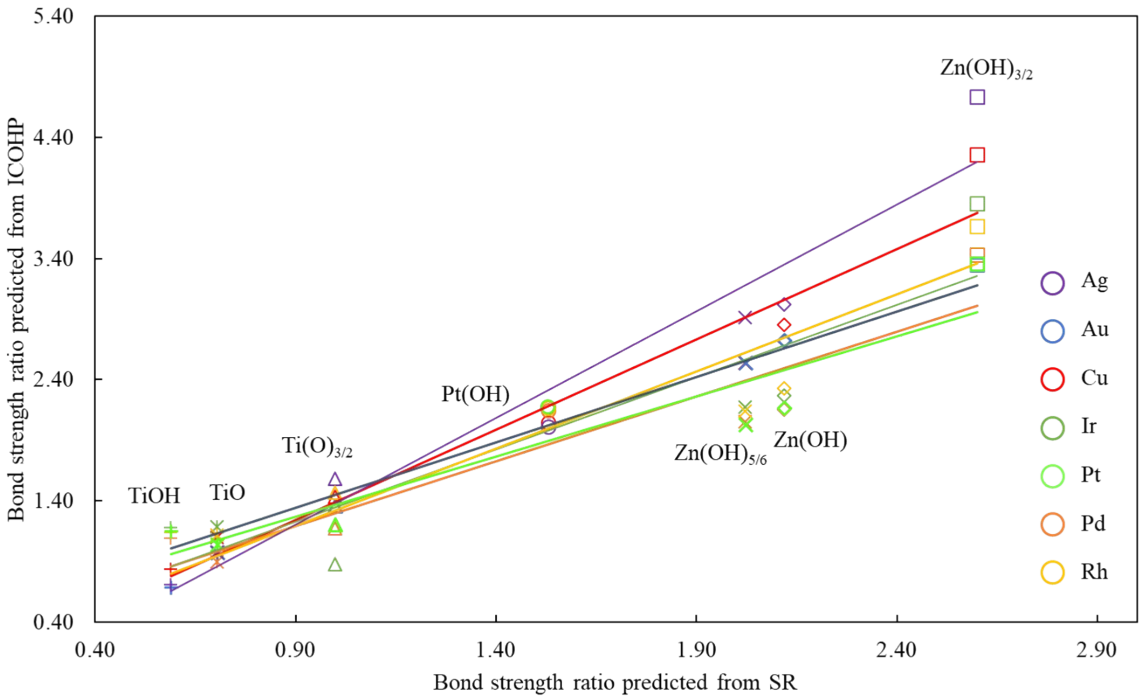

Figure S6 : The ratios of film to metal atom adsorption strengths calculated directly from ICOHP analysis (vertical axis) and estimated from DFT-calculated SR slopes (horizontal axis). The *TiOH, TiO,* $TiO_{3/2}$, *Pt(OH)*, $Zn(OH)_{5/6}$, *Zn(OH)*, and $Zn(OH)_{3/2}$ films are represented by plus, star,

triangle, circle, cross, diamond, and square markers, respectively. The red, orange, green, blue, purple, yellow, and light green points represent the Cu, Pd, Ir, Au, Ag, Rh, and Pt substrates respectively.

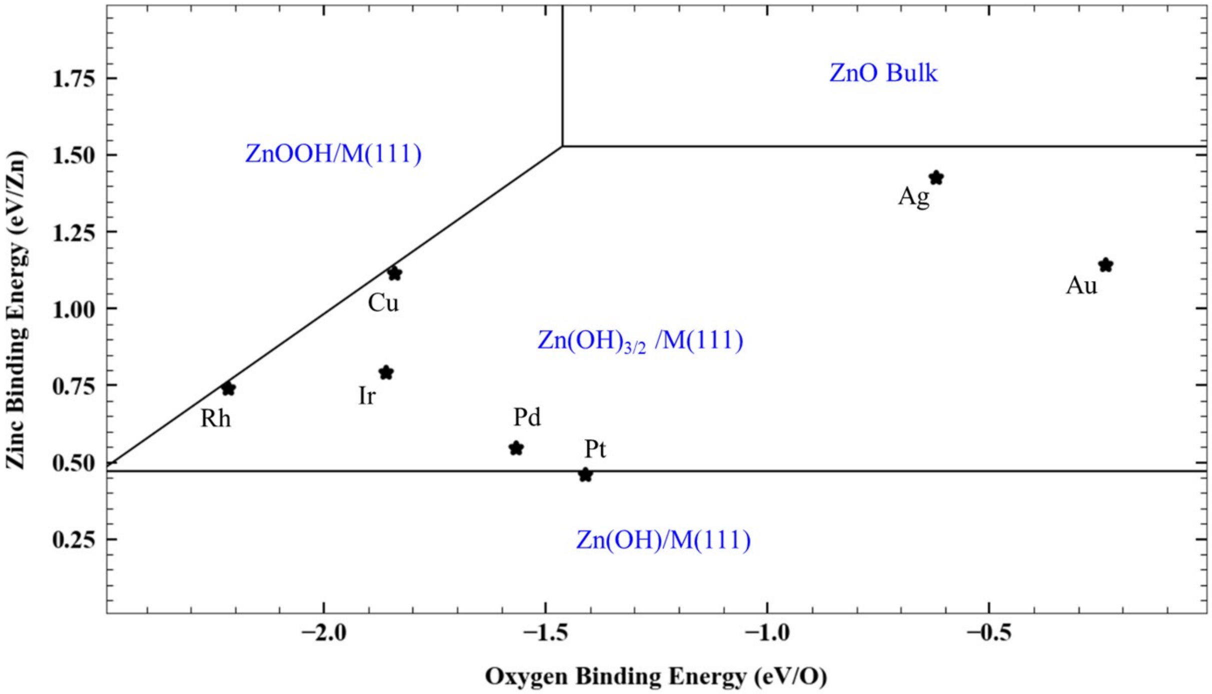

Figure S7 : Phase plots for ZnO_xH_y films on transition metal substrates with H_2 (g), O_2(g) (1 bar at 550K), and bulk ZnO. The oxygen binding energy and zinc binding energy are the independent descriptors. Scaling relationships described in the text are used to estimate the relevant film formation energies. The most stable structures for the given descriptor values are marked in blue. The black lines indicate the phase boundaries.

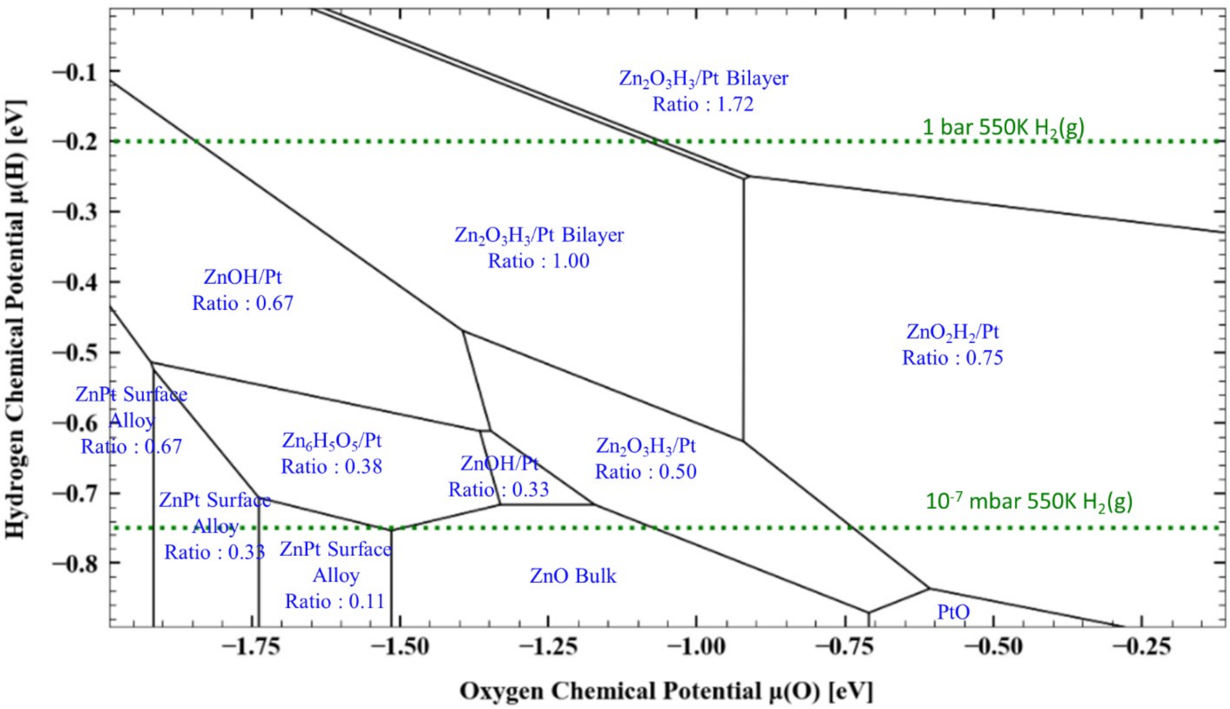

Figure S8 : Grand canonical phase diagram for ZnO_xH_x/Pt(111) with H_2 (g), O_2(g), and bulk ZnO as reference states. The dotted green lines represent the chemical potentials of H_2 at 1 bar, 550 K (top), and at 1×10^{-7} mbar, 550 K (bottom).
